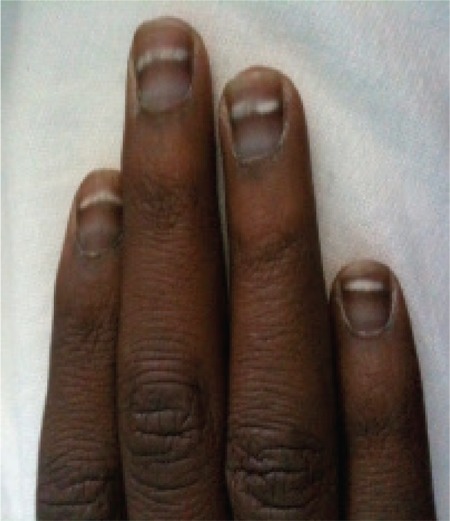# Mees’ Lines in an Acute Myeloid Leukemia Patient

**DOI:** 10.4274/Tjh.2013.0048

**Published:** 2013-09-05

**Authors:** Soumaya Anoun, Meryem Qachouh, Mouna Lamchahab, Asmae Quessar, Said Benchekroun

**Affiliations:** 1 Hopital du 20 Aout – Hematology & Pediatric Oncology Residence Fares 5 rue Abou Fares Bnou Hamdane, Casablanca, Morocco

A 26-year-old black male was treated for acute myeloid leukemia, subtype AML4. The karyotype showed trisomy of chromosome 8 and monosomy of chromosome 15. The patient received 5 courses of chemotherapy with complete remission after the first induction. Each aplasia was marked by deep infection, often treated with antibiotherapy and antifungal treatment. Chemotherapy courses included aracytine, daunorubicin, etoposide, and kidrolase. The patient developed multiple white lines on the fingernails, known as Mees’ lines [1,2]. The distance between the lines showed that the patient received the 2 last courses of chemotherapy with a large delay. Each line corresponds to the start of a chemotherapy course. Cytotoxic agents and infections can induce the temporary arrest of proliferation of the nail matrix, which is called Mees’ lines of the nail plate. The patient remains in complete remission with a follow-up of 6 months and the Beau’s lines of his nail plate have disappeared completely. Informed consent was obtained.

## CONFLICT OF INTEREST STATEMENT

The authors of this paper have no conflicts of interest, including specific financial interests, relationships, and/ or affiliations relevant to the subject matter or materials included.

## Figures and Tables

**Figure 1:**